# Endorectal Ultrasound Versus MRI for Lower and Middle Rectal Cancer Staging in Upfront Surgery: A Comparative Study

**DOI:** 10.3390/jcm15083039

**Published:** 2026-04-16

**Authors:** Riccardo Balestri, Silvia Strambi, Francesco Giudice, Mario Miccoli, Paola Vagli, Chiara Croce, Lucio Urbani, Niccolò Roffi, Francesco Arces, Piero Buccianti, Massimo Chiarugi, Dario Tartaglia

**Affiliations:** 1 General Surgery Unit, New Santa Chiara Hospital, 56124 Pisa, Italy; r.balestri@ao-pisa.toscana.it (R.B.); ccroce3@gmail.com (C.C.); l.urbani@ao-pisa.toscana.it (L.U.); nicolo.roffi@ao-pisa.toscana.it (N.R.); p.buccianti@ao-pisa.toscana.it (P.B.); 2 General and Emergency Unit & Trauma Center, New Santa Chiara Hospital, University of Pisa, 56124 Pisa, Italy; sil.strambi@gmail.com (S.S.); francesco.giudice98@gmail.com (F.G.); francesco.arces@gmail.com (F.A.); massimo.chiarugi@unipi.it (M.C.); 3 Statistical Department, Pisa University, 56124 Pisa, Italy; mario.miccoli2020@virgilio.it; 4 Cisanello Radiology Unit, Azienda Ospedaliero Universitaria Pisana, 56124 Pisa, Italy; paolavagli@yahoo.it

**Keywords:** transrectal ultrasound, magnetic resonance, rectal cancer, early stages

## Abstract

**Objectives**: This study aimed to compare the results obtained by endorectal ultrasound (ERUS) and magnetic resonance imaging (MRI) with the pathologic staging of the operative specimen in patients with lower and middle rectal cancer not treated with neoadjuvant therapy and undergoing surgery. **Methods**: From 2011 to 2022, all the consecutive patients with lower/middle rectal cancer who underwent surgery-first treatment were evaluated. The results of diagnostic examinations and the definitive pathological examination were considered and compared. **Results**: One hundred and one patients were enrolled in the study. The mean age was 72.5 (SD ± 9.7) years. F:M = 1:2. Mean distance from the anal orifice was 87 (SD ± 9.7) mm. Mean tumoral cranio-caudal extension was 35 (SD ± 12.5) mm. According to the T-stage, the κ coefficient showed a fair concordance between MRI and Pathology (κ = 0.294) and moderate between ERUS and Pathology (κ = 0.534). According to the N-stage, MRI was related to a lower concordance (κ = 0.138) than ERUS (κ = 0.337) with Pathology. Comparing ERUS with MRI, κ was higher in staging T (κ = 0.410), showing a moderate agreement. Stage N was related to a fair agreement between the two imaging methods (κ = 0.237). **Conclusions**: MRI and ERUS have similar results in performing the TN staging in patients with lower and middle rectal cancer who did not undergo neoadjuvant chemoradiotherapy. ERUS might be a valid option for staging patients who cannot have the possibility to perform an MRI.

## 1. Introduction

Accurate staging of rectal cancer should ideally determine the depth of invasion and presence of lymph node metastases and should ascertain the resectability of locally advanced tumors. A proper treatment strategy selection is based on the preoperative staging of the tumor. Assessment of tumor penetration through the rectal wall (T stage) represents a key determinant in selecting the most appropriate treatment strategy. Current international guidelines emphasize the role of imaging in stratifying patients and tailoring treatment according to tumor stage and associated risk factors. In locally advanced rectal disease, especially in tumors extending beyond the muscularis propria, neoadjuvant chemoradiotherapy is commonly recommended to improve resectability and reduce local recurrence [1,2]. Different methods for staging rectal cancer have been described: endorectal ultrasonography (ERUS), magnetic resonance imaging (MRI), and computed tomography (CT) [1]. MRI is widely used as the reference imaging modality for rectal cancer staging due to its ability to assess mesorectal structures and surgical margins, while ERUS offers significant advantages in evaluating early-stage tumors, particularly for assessing rectal wall infiltration and perirectal fat invasion. The integration of diffusion-weighted imaging (DWI) with MRI further enhances diagnostic performance by providing functional information about the tumor and adjacent mesorectal tissues [3,4]. Despite technological advancements, differentiating true tumor infiltration from desmoplastic reaction within the mesorectal fat remains challenging, potentially leading to staging inaccuracies and impacting clinical management. Moreover, all imaging techniques are associated with a non-negligible risk of overstaging, reported in up to 30% of cases in some series [5]. Although numerous studies have investigated the diagnostic performance of MRI and ERUS, results remain heterogeneous, especially in the assessment of intermediate stages such as T3 tumors, which are the most diagnosed. These discrepancies highlight the need for further comparative analyses in real-world clinical settings [6].

In addition to tumor depth, the assessment of lymph node involvement (N stage) represents a critical component of preoperative staging, as it significantly influences prognosis and therapeutic strategy. However, accurate nodal evaluation remains challenging with current imaging techniques. Both MRI and ERUS rely primarily on morphological criteria, such as nodal size, shape, border definition, and internal signal characteristics, which are inherently limited in distinguishing benign reactive nodes from metastatic involvement [7] MRI, particularly when combined with diffusion-weighted imaging (DWI), may improve the detection of suspicious lymph nodes, although its diagnostic accuracy remains variable. Similarly, ERUS can identify perirectal lymph nodes with high spatial resolution, but its performance is operator-dependent and restricted to the immediate perirectal region. As a result, both modalities show suboptimal sensitivity and specificity for N staging, often leading to under- or overstaging [8]. This limitation has important clinical implications, as inaccurate nodal assessment may affect the indication for neoadjuvant treatment and surgical planning. Therefore, improving the reliability of imaging-based nodal staging remains a key unmet need in rectal cancer management [9].

Due to their complex anatomy, tumors of the lower/middle rectum may pose important challenge in MRI staging. So far, a multimodal approach can provide a more accurate preoperative assessment, which is essential for treatment planning and patient prognosis [10].

The present study aimed to compare the staging results obtained by ERUS and MRI in patients with lower and middle rectal cancer not treated with neoadjuvant therapy and examine the grade of concordance between these procedures and the histology after the treatment.

## 2. Methods

### 2.1. Patient Selection

Patients with histologically proven diagnoses of adenocarcinoma of the rectum were evaluated from 2011 to 2022. Patients who underwent neoadjuvant chemotherapy, those with incomplete diagnostic examinations, those who had surgery outside our Institution, and neoplasia over 12 cm from the anal verge (because of the lower accuracy of ERUS) were excluded from the study. Staging exams were represented by computed tomography, magnetic resonance, and transrectal ultrasound. All patients were evaluated in a multidisciplinary oncologic group with an oncologist, a radiotherapist, a radiologist, and a surgeon. Imaging findings referred to clinical staging (cTN), while histopathological results to pathological staging (pTN). Age, sex, results of diagnostic examinations, and definitive pathological examination were considered. ERUS was performed preoperatively and interpreted by one rectal surgeon, with more than 50 examinations for rectal cancer performed per year. Neither the radiologist nor the ERUS operator knew the results of the counterpart procedure.

### 2.2. ERUS

Endorectal ultrasound (ERUS) was performed with 360° rotating probe 2052 Type with balloon (Pro Focus BK Medical sonographer, BK Medical, Trelleborg, Denmark). The frequencies of the linear array ranged from 5.5 to 10 MH. Using a standard syringe, the water standoff system was slowly filled with degassed water (max 200 mL). Three-dimensional EUS was used to observe the lesion’s location, size, and morphology, the degree of tumoral invasion of the rectal wall, the relationship between the lesion and the perirectal organs, and the extent of perirectal lymphadenopathy. EUS was performed preoperatively and interpreted by one rectal surgeon.

### 2.3. MRI

The imaging protocol was based on the minimum sequences recommended by the European Society of Gastrointestinal and Abdominal Radiology (ESGAR) guidelines, supplemented with additional sequences when necessary. MRI examinations were performed using a high-field 1.5/3 T scanner without contrast (Magnetom Symphony, Siemens Medical System, Erlangen, Germany).

The protocol included an axial T2-weighted fast spin-echo sequence (5 mm slice thickness) acquired in a plane orthogonal to the tumor to cover the entire pelvis, complemented by diffusion-weighted imaging (DWI) in the same axial plane (b-values: 0, 500, 1000) and corresponding apparent diffusion coefficient (ADC) maps.

After identifying the tumor’s location, orientation, and relevant lymph nodes (both mesorectal and extramesorectal), we performed thin-section (3 mm) axial-oblique T2-weighted sequences in a plane orthogonal to the tumor, followed by high-spatial-resolution T2-weighted coronal and sagittal images.

Rectal lesions and lymph nodes were evaluated using established imaging criteria outlined in the ESGAR guidelines, and findings were documented in a structured report (modified ESGAR template) [8]. Lymph node assessment was based on a combination of nodal size and morphology. Nodes with a short-axis diameter ≥ 9 mm or those displaying mucinous signal characteristics were considered suspicious. Smaller lymph nodes were classified as N+ only if they exhibited additional morphologically suspicious features, such as a round shape, indistinct borders, or heterogeneous signal.

### 2.4. Ultrasonographic Staging

The ultrasonographic staging of rectal cancer is as follows: uT1: tumor confined to the mucosa and submucosa; uT2: tumor invades the muscularis propria but does not reach the serosa; uT3: tumor extends through the muscularis propria into perirectal fat; uT4: tumor invades adjacent organs and tissues; uN0: no lymph node metastasis or prominent lymph nodes are noted around the rectum, or the lymph node diameter is less than 5 mm; uN+: lymph node metastasis.

Lymph nodes were classified as malignant if they met one of the following criteria: short axis > 9 mm or short axis 5–8 mm with at least 2 suspicious features or short axis < 5 mm with at least 3 suspicious features. The suspicious features were round hypoechoic shape, irregular borders, heterogeneous appearance [11].

All the exams were performed at least 15 days from the endoscopic biopsies.

### 2.5. Data Analysis

All the procedures evaluated: distance from the anal verge, size, depth of infiltration, presence of lymph nodes, infiltration of perirectal tissue. TNM classification was used for histological staging. The depth of transmural tumor invasion was assessed according to the 8th edition AJCC/TNM classifications for ERUS, MRI, and histopathologic examinations, and the results were compared retrospectively. A single expert pathologist evaluated all histological specimens.

### 2.6. Statistics

Descriptive statistics are reported as means, standard deviations, absolute frequencies, and percentages. Cohen’s kappa coefficient was used to evaluate the agreement between imaging modalities and histopathological findings, as it is a widely accepted measure of inter-method agreement beyond chance. Kappa values were interpreted according to standard benchmarks: <0.20 (poor agreement), 0.21–0.40 (fair agreement), 0.41–0.60 (moderate agreement), 0.61–0.80 (good agreement), and 0.81–1.00 (excellent agreement). Statistical analysis was carried out with XLSTAT 2020 and R 4.0.3 for Windows.

## 3. Results

**Figure 1:**
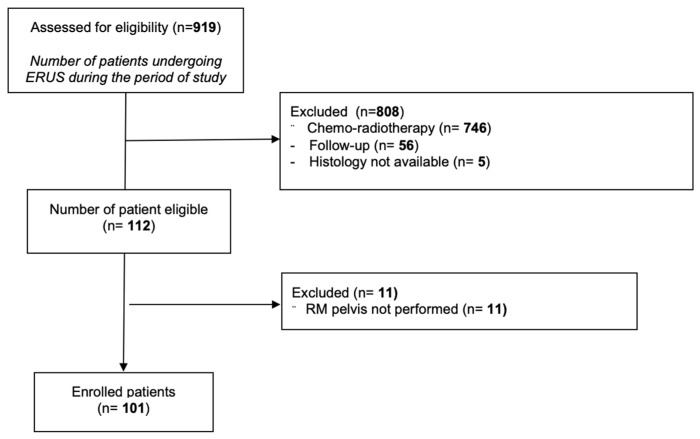
Flow chart of the patients enrolled in the study.

One hundred and one patients with rectal adenocarcinoma treated with a surgery-first treatment were enrolled in the study (Figure 1). The mean age was 72.5 (SD ± 9.7) years. F:M = 1:2. Mean distance from the anal orifice was 87 (SD ± 9.7) mm. Mean tumoral cranio-caudal extension was 35 (SD ± 12.5) mm. MRI was performed with a 1.5 and a 3 Tesla scanner in 34 and 67 patients, respectively. Considering clinical imaging staging (cTN), MRI classified tumors as cT0 (*n* = 10), cT1 (*n* = 7), cT2 (*n* = 17), cT3 (*n* = 56), and cT4 (*n* = 11), with nodal involvement (cN+) observed in 45 patients. ERUS staging showed uT0 (*n* = 19), uT1 (*n* = 6), uT2 (*n* = 16), uT3 (*n* = 51), and uT4 (*n* = 9), with uN+ identified in 54 patients. Pathological staging (pT) of the primary rectal tumor, evaluated after surgical resection, comprehended: pT0: 17 patients; pT1: 12 patients; pT2: 22 patients; pT3: 48 patients; pT4: 2 patients. Lymph node involvement (pN+) was present in 31 out of 101 patients.

The results obtained by each imaging modality in tumor stage assessment are compared with the pathological findings in Table 1 (staging parameter T) and Table 2 (staging parameter N). MRI and ERUS were evaluated and compared for each stage in Table 3. According to the T-stage, the κ coefficient showed a fair concordance between MRI and Pathology (κ = 0.294) and moderate concordance between ERUS and Pathology (κ = 0.534). According to the N-stage, MRI was related to a lower concordance (κ = 0.138) than ERUS (κ = 0.337) with Pathology. Comparing ERUS with MRI, κ was higher in staging T (κ = 0.410), showing a moderate agreement. Stage N was related to a fair agreement between the two imaging methods (κ = 0.237).

## 4. Discussion

Although the total incidence of rectal cancer has been decreasing, the incidence is increasing (from 1.8 cases per 100,000 people in 1985 to 3.2 per 100,000 in 2018), mainly in patients under 50 years old. It is predicted to nearly double by 2030 in younger patients (20–30 years old) [12,13]. Currently, available treatments can cause important dysfunctions (i.e., infertility, urinary and defecatory issues) that could be more psychologically and socially burdensome in younger patients [14]. An early and exact pathology staging is crucial for choosing adequate therapy and balancing survival and side effects.

The cornerstone treatment of early and advanced stage rectal cancer is the total mesorectal excision (TME) as described by R. J. Heald et al. in 1982 [15]. This surgery consists of the excision of the rectum and the tumor with all its mesenteric blood and lymphatic supply, as far as the point of emergence of the anorectum, demonstrated to prevent local recurrence of the tumor. Previously, in locally advanced rectal cancer, surgery was used to be followed by adjuvant radiotherapy (with or without chemotherapy). The use of adjuvant therapy in locally advanced tumors had already been questioned in the late 90s. Merchant et al. showed that T3N0M0 lesions exclusively treated with surgery in selected patients had only 9% local recurrence [16]. In 2004, it was proven that adjuvant chemoradiotherapy led to higher rates of local failure (10.1% vs. 7.1%) and higher rates of acute and late high-grade toxicities (respectively 40% vs. 27% and 24% vs. 14%) rather than neoadjuvant therapies without significant differences in ten years survival rate [17]. Therefore, neoadjuvant chemoradiotherapy became the standard treatment for stage II and III tumors. Adequate preoperative imaging is fundamental. However, it still conceals many difficulties in clinical practice [18].

In the following years, research focused on accurate preoperative therapy: in particular, the Mercury study group (2011) demonstrated the possibility of identifying good prognosis tumors with high-resolution and thin-section MRI that could be treated with surgery alone with a local failure rate of 3% [19]. These tumors with a good prognosis were identified as T2/T3a-b lesions (=Tumor Extramural Depth of Invasion less than or equal to 5 mm) with predicted safe circumferential resection margins, and this was possible by the already proven sub-staging accuracy of MRI compared with histology staging (calculated equivalent to within 0.5 mm) in 2007 [20]. The analysis of our study population confirmed that most rectal cancers are clinically diagnosed at the T2 (22%) and T3 stage (48%).

Endorectal sonography is widely accepted as a valuable imaging tool for staging rectal cancer. ERUS is widely recommended for the evaluation of early rectal cancer (cT1–cT2), where its high spatial resolution allows a detailed assessment of rectal wall layers [4,21]. It can identify the T stage (ranging from 88% to 95%) and recognize early vs. advanced tumors with high accuracy (94%) [21]. Furthermore, endorectal ultrasound better identifies the circumferential resection margin than MRI [7,22,23,24,25]. Our results showed lower accuracy in identifying T-stage for MRI (k = 0.294) than ERUS (k = 0.534).

The concordance between MRI and pathology findings of lymph node metastasis (57%) was slightly less than what was found in the literature [26]; conversely, the concordance between ERUS and pathology findings was slightly higher [27]. It is worth noting that there is high heterogeneity in results regarding ERUS accuracy for the assessment of rectal cancer, showing higher accuracy in older studies and in studies with fewer patients [28]. The MRI overstaging of T2–T3 lesions observed in our cohort may be explained by the difficulty in distinguishing true tumor infiltration from desmoplastic reaction or peritumoral fibrosis. This limitation may lead to an overestimation of tumor depth, particularly in intermediate stages. Similarly, ERUS may overestimate tumor invasion, especially in T2–T4 lesions, where peritumoral inflammation or fibrosis can be misinterpreted as deeper wall infiltration.

The choice of imaging modality may also be influenced by availability, cost, and operator expertise. While MRI provides a comprehensive evaluation, ERUS is more accessible but operator-dependent. These factors should be considered when selecting the optimal staging strategy.

From a clinical perspective, the differences observed between MRI and ERUS performance may reflect their intrinsic technical characteristics. MRI provides a comprehensive evaluation of the pelvis, including mesorectal fascia involvement and extramural vascular invasion, which are crucial for surgical planning. However, its spatial resolution may be insufficient to accurately discriminate early tumor infiltration layers, potentially explaining the lower concordance observed in T staging in our series. On the other hand, ERUS allows a detailed visualization of the rectal wall architecture, making it particularly suitable for early-stage disease. This may account for the higher agreement with pathology observed in our cohort. Nevertheless, ERUS is inherently operator-dependent and limited in the evaluation of stenotic or high rectal lesions, which restricts its universal applicability. The relatively low concordance observed for nodal staging is consistent with the previous literature, confirming that lymph node assessment remains a major limitation of current imaging modalities. Morphological criteria alone are often insufficient to distinguish reactive from metastatic lymph nodes, and size-based thresholds may lead to both under- and overstaging. An additional relevant aspect is the potential complementary role of MRI and ERUS. Rather than considering these techniques as mutually exclusive, their combined use could improve diagnostic accuracy in selected cases, particularly when imaging findings are equivocal or when treatment decisions rely on precise T sub-staging. From a practical standpoint, our findings suggest that ERUS may represent a valuable alternative in settings where MRI is not available or contraindicated. This is particularly relevant in resource-limited environments, where access to high-quality MRI may be restricted.

Future research should focus on integrating advanced imaging techniques, including radiomics and artificial intelligence-based analysis, which may enhance staging accuracy and reduce interobserver variability. Prospective multicenter studies are also warranted to validate these findings and define standardized diagnostic pathways.

The limitation of this study is the retrospective nature of the analysis. Moreover, while the same operator performed all ERUS, MRI was executed by different radiologists, possibly resulting in more variability of reports. Furthermore, no comparisons were made in terms of distance from the anal verge and tumoral size, as not focused for the aim of the study. However, the present study gives an overview of managing different rectal tumoral stages in a large cohort of patients. From a prospective point of view, if the data were confirmed on a larger cohort of patients, ERUS might represent a correct choice in staging process, with advantages in terms of costs and precision, whereas MRI is not available.

## 5. Conclusions

MRI and ERUS have similar results in performing TN staging for patients with lower-middle rectal cancer who do not undergo neoadjuvant chemoradiotherapy. ERUS might be a valid option for staging patients who cannot have the possibility to perform an MRI.

## Figures and Tables

**Table 1:** Comparison of different preoperative imaging modalities with the post-surgical pathology results according to the staging parameter T. (**A**): Pathology-Stage + MRI-Stage; (**B**): Pathology-Stage + ERUS-Stage. Percentages are calculated by column, representing the proportion of cases within each pathological stage.

A					
	Pathology-Stage (pT)				
MRI-Stage (cT)	T0	T1	T2	T3	T4
T0	8 (47%)	1 (8%)	1 (5%)	0	0
T1	3 (18%)	2 (17%)	2 (9%)	0	0
T2	3 (18%)	4 (33%)	6 (27%)	4 (8%)	0
T3	3 (18%)	5 (42%)	11 (50%)	36 (75%)	1 (50%)
T4	0	0	2 (9%)	8 (17%)	1 (50%)
***Cohen’s kappa*** = 0.294					
**B**					
	**Pathology-Stage (pT)**				
**ERUS-Stage (uT)**	**T0**	**T1**	**T2**	**T3**	**T4**
T0	14 (82%)	3 (25%)	2 (9%)	0	0
T1	0	6 (50%)	0	0	0
T2	3 (18%)	1 (8%)	10 (45%)	1 (2%)	0
T3	0	2 (17%)	9 (41%)	38 (81%)	2 (100%)
T4	0	0	1 (5%)	8 (17%)	0
***Cohen’s kappa*** = 0.534					

**Table 2:** Comparison of different preoperative imaging modalities with the post-surgical pathology results according to the staging parameter N. (**A**): Pathology-Stage + MRI-Stage; (**B**): Pathology-Stage + ERUS-Stage. Percentages are calculated by column, representing the proportion of cases within each pathological stage.

A		
	Pathology-Stage (pN)	
MRI-Stage (cN)	N0	N+
N0	25 (68%)	31 (44%)
N+	12 (32%)	33 (52%)
***Cohen’s kappa*** = 0.170		
**B**		
	**Pathology-Stage (pN)**	
**ERUS-Stage (uN)**	**N0**	**N+**
N0	27 (73%)	20(31%)
N+	10 (27%)	44 (69%)
***Cohen’s kappa*** = 0.400		

**Table 3:** Comparison between MRI-Stage and ERUS-Stage. (**A**): T stage. (**B**): N-stage. Percentages are calculated by column.

A					
	MRI-Stage (cT)				
ERUS-Stage (uT)	T0	T1	T2	T3	T4
T0	10 (100%)	4 (57%)	3 (18%)	2 (4%)	0
T1	0	1 (14%)	3 (18%)	2 (4%)	0
T2	0	1 (14%)	6 (35%)	8 (15%)	0
T3	0	1 (14%)	3 (18%)	40 (73%)	7 (64%)
T4	0	0	2 (18%)	3 (5%)	4 (36%)
***Cohen’s kappa*** = 0.410					
**B**					
			**MRI-Stage (cN)**		
**ERUS-Stage (uN)**			**N0**	**N+**	
N0			27 (52%)	14 (28%)	
N+			25 (48%)	35 (72%)	
***Cohen’s kappa*** = 0.237					

## Data Availability

The datasets generated during and/or analyzed during the current study are available from the corresponding author on reasonable request.

## Abbreviations

ERUS	endorectal ultrasonography
MRI	magnetic resonance imaging
DWI	diffusion-weighted imaging
ESGAR	European Society of Gastrointestinal and Abdominal Radiology
ADC	apparent diffusion coefficient
TME	total mesorectal excision
AJCC/TNM	American Joint Committee on Cancer/Tumor-Nodes-Metastasis
